# Supplementary material for: Degradation of Organic Methyl Orange (MO) Dye Using a Photocatalyzed Non-Ferrous Fenton Reaction
Source: Nanomaterials (Basel). 2023 Feb 6;13(4):639. doi: 10.3390/nano13040639 (PMC9965019; doi:10.3390/nano13040639)
Supplement: Supplementary file 1 [file nanomaterials-13-00639-s001.zip › nanomaterials-2153675-supplementary.pdf]

Supplementary Materials

Degradation of Organic Methyl Orange (MO) Dye Using Photocatalyzed Non-Ferrous Fenton Reaction

Sifani Zavahir[1], Tasneem Elmakki[1], Nourhan Ismail[1], Mona Gulied[1], Hyunwoong Park[2], Dong Suk Han[1,3,*]

[1] Center Advanced Materials (CAM), Qatar University, P.O. Box 2713, Doha, Qatar
[2] School of Energy Engineering, Kyungpook National University, Daegu 41566, Republic of Korea
[3] Department of Chemical Engineering, College of Engineering, Qatar University, P.O. Box 2713, Doha, Qatar
* Correspondence: dhan@qu.edu.qa; Tel.: +974-4403-5686

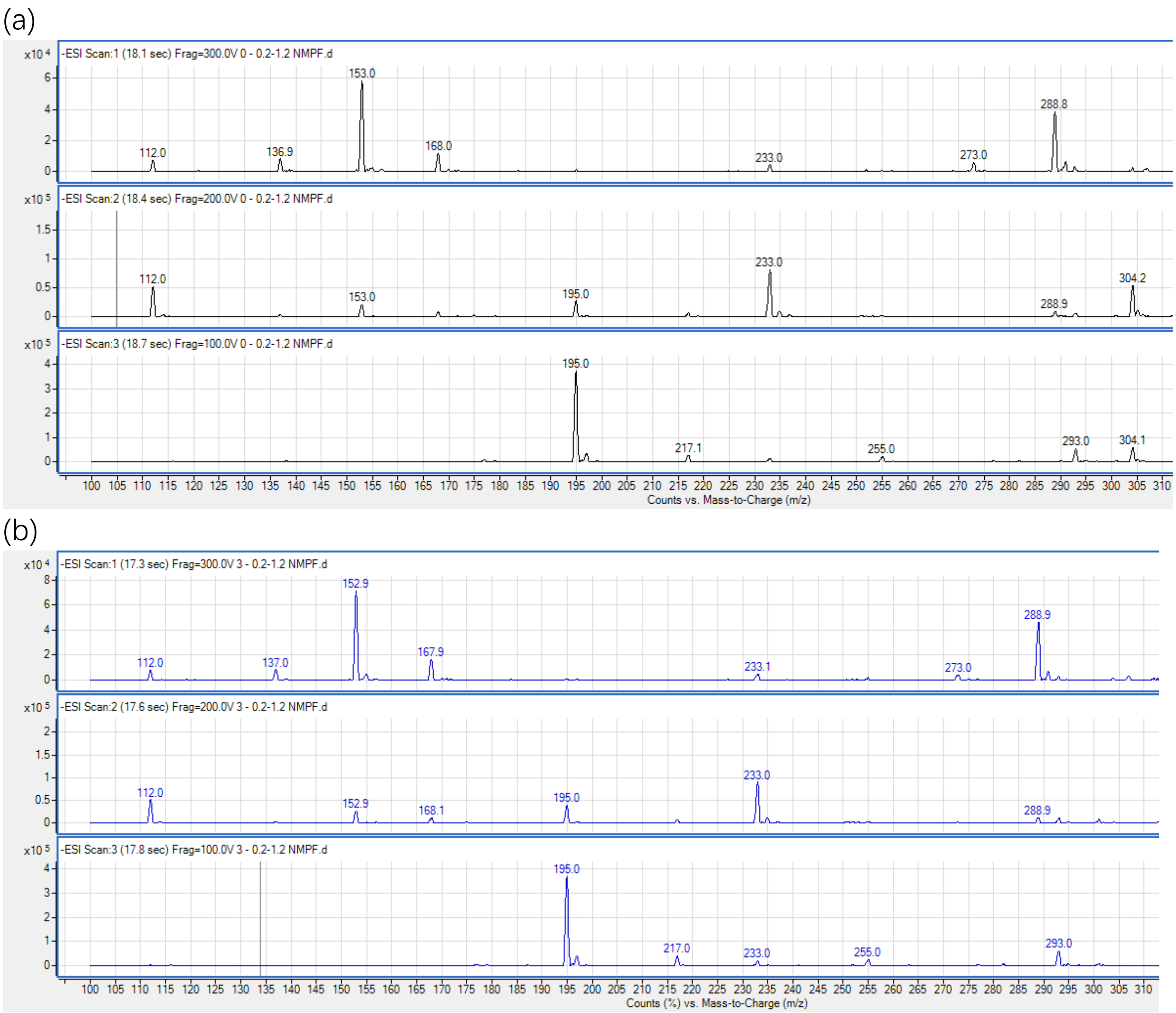

Figure S1. The LC/MS/MS chromatograms of the reaction solution at two different time intervals. The presence of methyl orange dye at m/z 304 (a) after 20 minutes of the experiment and (b) after 40 minutes.
